# Supplementary material for: Identification and Analysis of the Expression of the PIP5K Gene Family in Tomatoes
Source: Int J Mol Sci. 2023 Dec 21;25(1):159. doi: 10.3390/ijms25010159 (PMC10778592; doi:10.3390/ijms25010159)
Supplement: Supplementary file 1 [file ijms-25-00159-s001.zip › ijms-2739114-supplementary.pdf]

Supplementary Materials

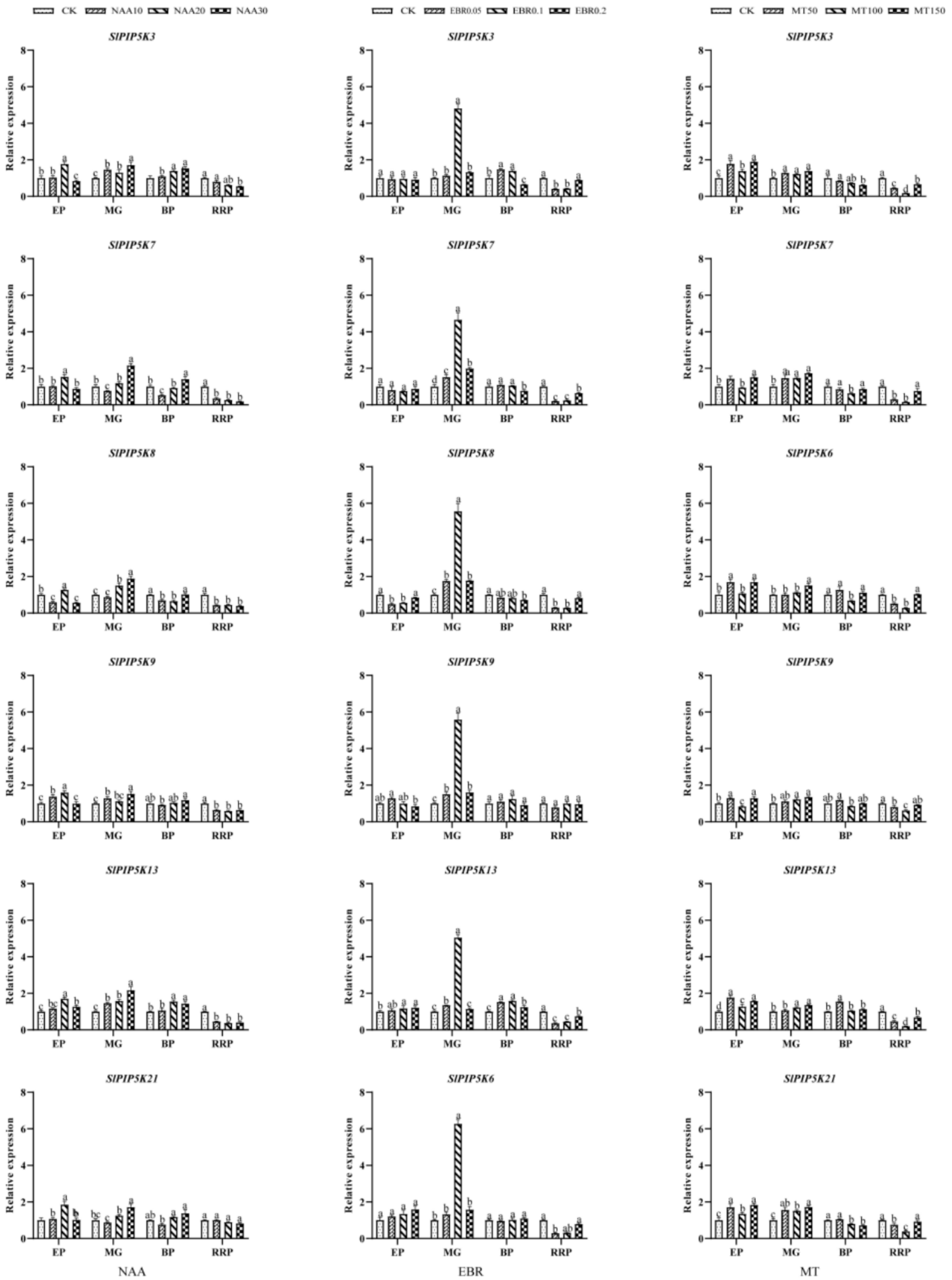

Figure S1. Expression of tomato *SlPIP5K* genes under different hormones. EP: swelling stage; MG: green ripening stage; BP: color conversion stage; RRP: red ripening stage. Different lower-case letters indicate significant differences between means as measured by ANOVA followed by Duncan's multiple range test ($p < 0.05$).
